# Endovascular coiling vs. surgical clipping for ruptured intracranial aneurysms: an in-hospital outcome win ratio analysis from a Colombian tertiary center

**DOI:** 10.3389/fradi.2025.1684496

**Published:** 2025-12-12

**Authors:** Santiago Quiceno-Ramírez, Enrique Carlos García-Pretelt, Valentina Mejía-Quiñones, Edgar Folleco-Pazmiño

**Affiliations:** 1 Centro de Investigaciones Clínicas, Fundación Valle del Lili, Cali, Colombia; 2 Radiólogo, Departamento de Radiología e Imágenes Diagnósticas, Fundación Valle del Lili, Cali, Colombia; 3 Facultad de Ciencias de la Salud, Fundación Valle del Lili, Cali, Colombia; 4 Neuroradiólogo Intervencionista, Departamento de Intervencionismo Vascular, Fundación Valle del Lili, Cali, Colombia

**Keywords:** intracranial aneurysm, subarachnoid hemorrhage, endovascular coiling, surgical clipping, win ratio, outcomes research, Latin America

## Abstract

**Background:**

The optimal management approach for ruptured intracranial aneurysms remains debated, with limited real-world evidence from Latin American populations. This study compared in-hospital outcomes between endovascular coiling and surgical clipping using a hierarchical win ratio (WR) analysis.

**Methods:**

We conducted a single-center retrospective cohort study of 194 patients with ruptured intracranial aneurysms treated at a tertiary referral center (2011–2022). Patients were treated with either endovascular coiling (*n* = 73) or surgical clipping (*n* = 121). The primary outcome was the win ratio, analyzing a hierarchical composite endpoint of: (1) in-hospital mortality, (2) unfavorable functional outcome at discharge (modified Rankin Scale >2), (3) major complications, and (4) prolonged ICU stay (>10 days). Secondary analyses included multivariable logistic regression and prespecified subgroup analyses by clinical severity and aneurysm location.

**Results:**

Baseline measured characteristics were balanced between groups. The win ratio significantly favored endovascular coiling (WR 1.75, 95% CI: 1.67–1.84, *p* < 0.001), indicating 75% more wins in the hierarchical outcome comparison. All individual components significantly favored coiling: mortality (WR = 1.35, *p* < 0.001), unfavorable functional outcome (WR = 1.53, *p* < 0.001), major complications (WR = 1.70, *p* < 0.001), and prolonged ICU stay (WR = 1.25, *p* < 0.001). Benefits were consistent across subgroups, including Hunt & Hess grades I-II (WR = 2.00) and III-V (WR = 1.96), and across most aneurysm locations. In contrast, multivariate logistic regression for poor outcome showed a favorable but non-significant trend for coiling (OR = 0.55, *p* = 0.102), while confirming Hunt & Hess ≥3 (OR = 5.54, *p* < 0.001) and modified Fisher ≥3 (OR = 3.85, *p* = 0.044) as dominant prognostic factors.

**Conclusion:**

In this Colombian cohort, hierarchical outcome analysis suggested superior in-hospital outcomes for endovascular coiling vs. surgical clipping. However, the substantial attenuation of this association in adjusted analyses indicates that these apparent advantages may largely reflect case selection patterns rather than inherent treatment superiority, as residual confounding by aneurysm complexity cannot be excluded.

## Introduction

The management of ruptured intracranial aneurysms, the leading cause of spontaneous subarachnoid hemorrhage (SAH), remains a critical challenge in neurovascular medicine (1). This life-threatening event is associated with high rates of morbidity and mortality, demanding prompt and effective intervention to secure the aneurysm and prevent rebleeding (2). For decades, the cornerstone of treatment was microsurgical clipping, a procedure that involves a craniotomy to place a clip across the aneurysm neck. However, the landmark International Subarachnoid Aneurysm Trial (ISAT) in 2002 fundamentally shifted this paradigm (3). This randomized controlled trial demonstrated that in patients with ruptured aneurysms suitable for either therapy, endovascular coiling resulted in a significantly higher rate of independent survival at one year compared to neurosurgical clipping.

Subsequent long-term follow-up from ISAT and other studies have refined our understanding of this comparison. While the survival and functional outcome advantage for coiling persists for up to 10 years, it is tempered by a higher long-term risk of rebleeding from the treated aneurysm, underscoring a trade-off between initial procedural safety and long-term durability (4, 5). The most recent Cochrane systematic review, which synthesizes all available randomized evidence, concludes with moderate-quality evidence that endovascular coiling is associated with better functional outcomes at one year, reinforcing its role as the preferred first-line treatment when feasible (6).

Despite this robust evidence base, several important gaps and nuances persist. The majority of evidence, including that from ISAT, is derived from populations where patients were predominantly in good clinical grade and had aneurysms located in the anterior circulation, raising questions about the generalizability of these findings to all patient profiles (4, 6). Furthermore, the comparison is often based on conventional statistical methods that may not fully capture the hierarchical nature of clinical outcomes, where mortality is prioritized over morbidity, and functional status over hospital stay. The win ratio methodology, which employs a pre-specified hierarchical composite endpoint, offers a more patient-centered and clinically nuanced assessment of treatment benefit, but its application in neurovascular research, particularly in real-world settings, remains limited (7).

Finally, there is a notable scarcity of data from Latin American populations. Local factors, including healthcare system structures, resource availability, and genetic or epidemiological characteristics, may influence treatment outcomes and the general applicability of international guidelines. Therefore, it is crucial to evaluate the comparative effectiveness of these two pivotal interventions within this specific context.

This study aims to compare outcomes between endovascular coiling and surgical clipping for ruptured intracranial aneurysms in a Colombian tertiary care center. Utilizing the novel win ratio analysis to provide a comprehensive assessment of treatment benefit across a hierarchy of clinically relevant endpoints, we seek to contribute valuable real-world evidence from a Latin American setting to the ongoing global discourse on optimal aneurysm management.

## Methods

### Study design and population

We conducted a single-center, retrospective cohort study of patients treated at our tertiary referral center between January 2011 and December 2022. Initial screening identified 620 patients through ICD-10 diagnosis codes and procedure codes related to cerebrovascular interventions.

Inclusion criteria comprised: (1) adult patients (≥18 years), (2) angiographically confirmed ruptured intracranial aneurysm, defined as Hunt & Hess grade I-V or modified Fisher grade ≥1, and (3) treatment with either endovascular coiling or surgical clipping.

Exclusion criteria were: (1) unruptured aneurysms, (2) other cerebrovascular diagnoses including arteriovenous malformations, cavernomas, and non-aneurysmal subarachnoid hemorrhage, (3) non-aneurysmal intracranial pathologies, and (4) combined treatments or lack of complete outcome data.

### Variables and data sources

Data were obtained through retrospective review of electronic medical records and image files from the institutional PACS system. Demographic variables included age and sex. Initial clinical severity was assessed using the Hunt & Hess and modified Fisher scales. Aneurysm characteristics included location, maximum aneurysm diameter, and neck width. Follow-up was performed until hospital discharge.

### Interventions

Treatment allocation was decided by a multidisciplinary team that considered aneurysm characteristics, patient clinical condition, and anatomical factors. Endovascular therapy consisted of coil embolization via selective catheterization until complete occlusion or until procedural safety limits were reached. Open surgery involved standard microsurgical clipping via a transcranial approach. The study focused specifically on the comparison between coil embolization and surgical clipping, excluding flow diversion techniques or other advanced procedures.

### Outcome variables

The primary outcome was WR, analyzing a predefined hierarchical composite endpoint that considered: (1) in-hospital mortality, (2) unfavorable functional outcome (modified Rankin scale >2 in survivors), (3) major complications, defined as a binary variable that included rebleeding, confirmed cerebral ischemia, symptomatic vasospasm, or hydrocephalus requiring shunting, and (4) prolonged ICU stay (>10 days in survivors).

Secondary endpoints included individual component analyses and traditional logistic regression for poor outcome (mRS >2, including death).

### Statistical analysis

The WR was calculated by pairwise comparison of all patients between treatment groups, assigning wins according to the predefined hierarchy. Statistical significance was determined using binomial tests. Comprehensive sensitivity analyses were performed that included different hierarchy orders.

Multivariate logistic regression analysis adjusted for age, sex, Hunt & Hess scale, modified Fisher scale, maximum aneurysm diameter, and neck width. Subgroup analyses were prespecified for clinical severity strata and aneurysm location.

Given anatomical and surgical similarities, anterior cerebral artery (ACA) and anterior communicating artery (ACoA) aneurysms were analyzed as a combined “anterior circulation” subgroup.

All analyses were performed using RStudio, considering statistical significance at *p* < 0.05.

### Ethical considerations

The study was approved by the institutional ethics committee and was conducted in accordance with the principles of the Declaration of Helsinki. Given the retrospective design, exemption from individual informed consent was obtained.

## Results

### Baseline characteristics and outcomes

**Figure 1:**
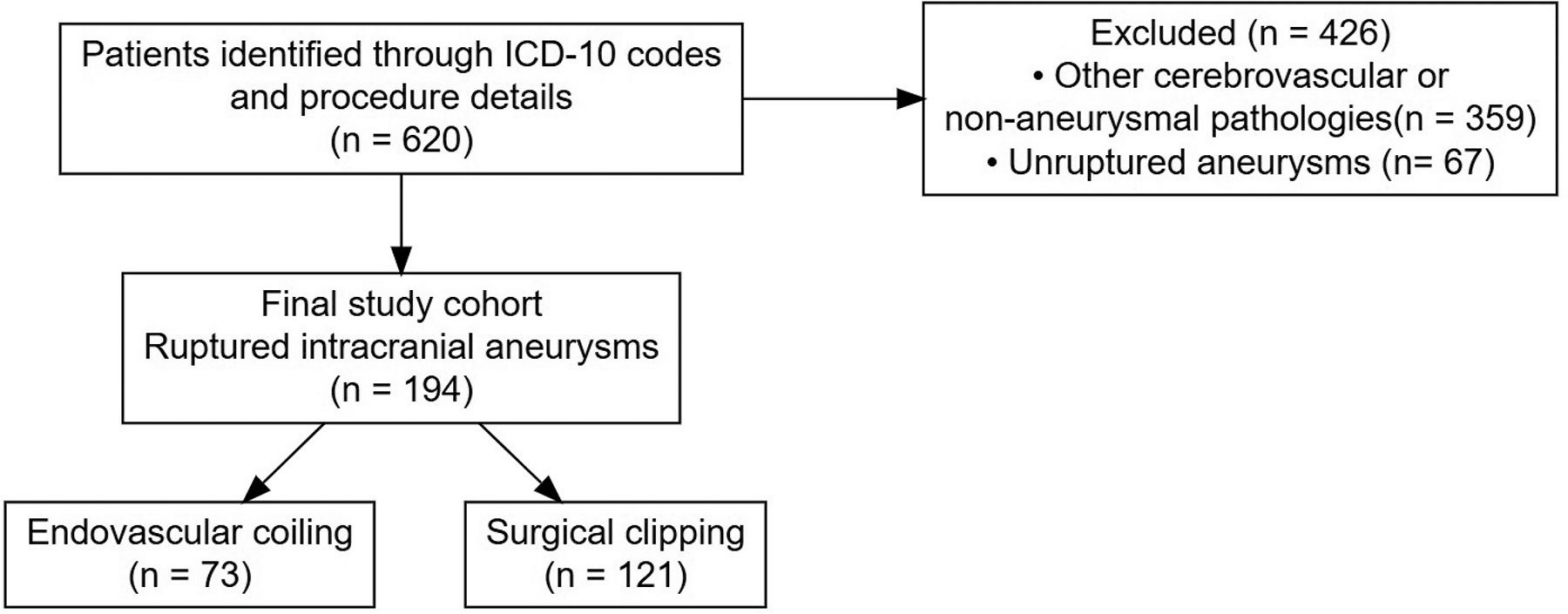
Patient selection flowchart.

The patient selection process is summarized in Figure 1. From an initial pool of 620 patients screened through ICD-10 codes, 426 were excluded based on predefined criteria, resulting in a final cohort of 194 patients with angiographically confirmed ruptured intracranial aneurysms. The treatment distribution was 73 (37.6%) endovascular coiling and 121 (62.4%) surgical clipping.

Baseline characteristics were balanced between groups, with a mean age of 55.3 ± 12.9 years and 28.9% male patients. Distribution by initial severity showed 46.9% with Hunt & Hess ≥3 and 82.5% with severe modified Fisher (≥3), with no significant differences between treatment groups. The most common location for both groups was the internal carotid artery (39.2%).

Regarding outcomes, ICU and hospital length of stay (LOS) median was longer in the endovascular coiling group, without reaching statistical significance (*p* = 0.07). No significant differences were found between groups for death or functional outcome (*p* = 0.479 and 0.167, respectively) (Table 1).

**Table 1:** Baseline characteristics and discharge functionality.

Variable	Total	Endovascular coiling	Open surgery	*p*-value
N	194	73	121	
Age, years ± SD	55.3 ± 12.9	54.3 ± 13.0	56.0 ± 12.9	0.388
Male sex, %	56 (28.9%)	27 (37.0%)	29 (24.0%)	0.076
Hunt & Hess ≥3, %	91 (46.9%)	33 (45.2%)	58 (47.9%)	0.826
mFisher ≥3, %	160 (82.5%)	63 (86.3%)	97 (80.2%)	0.371
Location, %				0.084^a^
Anterior cerebral artery	16 (8.2%)	8 (11.0%)	8 (6.6%)	
Anterior communicating artery	46 (23.7%)	16 (21.9%)	30 (24.8%)	
Internal carotid artery	76 (39.2%)	23 (31.5%)	53 (43.8%)	
Middle cerebral artery, M1	29 (14.9%)	17 (23.3%)	12 (9.9%)	
Middle cerebral artery, M2	10 (5.2%)	5 (6.8%)	5 (4.1%)	
Basilar artery	3 (1.5%)	0 (0.0%)	3 (2.5%)	
Other	14 (7.2%)	4 (5.5%)	10 (8.3%)	
Maximum aneurysm diameter, mm ± SD	6.6 ± 4.1	6.1 ± 3.2	6.9 ± 4.5	0.144
Neck diameter, mm ± SD	3.1 ± 1.4	3.1 ± 1.5	3.2 ± 1.2	0.643
Outcomes				
ICU LOS in survivors, days [IQR]	13.0 [9.0–16.0]	14.0 [10.0–16.0]	12.0 [9.0–15.0]	0.072
Hospital LOS in survivors, days [IQR]	14.0 [11.0–21.0]	15.0 [11.0–26.0]	14.0 [11.0–19.0]	0.107
Death, mRS 6	44 (22.7%)	19 (26.0%)	25 (20.7%)	0.479
Favorable, mRS 0–2	122 (62.9%)	41 (56.2%)	81 (66.9%)	0.167
Unfavorable, mRS 3–5	27 (13.9%)	12 (16.4%)	15 (12.4%)	

SD, standard deviation; mFisher, modified Fisher; IQR, interquartile range; mRS, modified Rankin Score.

a Fisher's Exact Test due to low frequencies in some categories.

### Primary and subgroup analysis

The primary WR analysis demonstrated a significant superiority of endovascular therapy over open surgery in hierarchical analysis, with a WR of 1.75 (95% CI: 1.67–1.84, *p* < 0.001) (Table 2).

**Table 2:** Win ratio of primary outcome, individual components and subgroup analysis.

Variable	*n*	WR	CI 95%	*p*-value
Primary outcome				
Global WR	194	1.75	1.67–1.84	<0.001
Individual components				
Mortality	194	1.35	1.26–1.45	<0.001
Unfavorable mRS (≥3), in survivors		1.53	1.41–1.67	<0.001
Major complications (ischemia, vasospasm, hydrocephalus), in survivors		1.70	1.60–1.81	<0.001
ICU LOS >10 d, in survivors		1.25	1.18–1.32	<0.001
Subgroup analysis				
Hunt & Hess III-V	91	1.96	1.76–2.18	<0.001
Hunt & Hess I-II	103	2.00	1.82–2.19	<0.001
mFisher 3–4	160	1.31	1.24–1.39	<0.001
mFisher 1–2	34	14.14	8.22–24.32^†^	<0.001
Location analysis				
Internal carotid artery	76	1.67	1.47–1.90	<0.001
Anterior circulation subgroup (AComA + ACA)	62	1.34	1.16–1.54	<0.001
Middle cerebral artery, M1	29	2.52	1.82–3.50	<0.001
Middle cerebral artery, M2	10	2.50	0.97–6.44	0.078

WR, win ratio; CI, confidence interval; mRS: modified Rankin Score; ICU: intensive care unit; LOS: length of stay; mFisher: modified Fisher; AComA: anterior communicating artery; ACA: anterior cerebral artery.

† Extreme WR likely due to complete separation in small subgroup (*n* = 34).

When examining the individual components of the hierarchy, all consistently favored endovascular therapy. Mortality showed a WR of 1.35 (95% CI: 1.26–1.45, *p* < 0.001), while unfavorable functional outcome in survivors (mRankin ≥3) had a WR of 1.53 (95% CI: 1.41–1.67, *p* < 0.001). Major complications were significantly lower in the endovascular group (WR 1.70, 95% CI: 1.60–1.81, *p* < 0.001), and prolonged ICU stay also favored this group (WR 1.25, 95% CI: 1.18–1.32, *p* < 0.001).

The superiority of endovascular therapy remained consistent across all prespecified subgroups. In patients with mild Hunt & Hess (I-II), the WR was 2.00 (95% CI: 1.82–2.19, *p* < 0.001), while in those with severe Hunt & Hess (III-V) it was 1.96 (95% CI: 1.76–2.18, *p* < 0.001). The subgroup with mild subarachnoid hemorrhage (Fisher 1–2) showed a particularly marked benefit (WR 14.14, 95% CI: 8.22–24.32, *p* < 0.001), although this should be interpreted with caution given the limited sample size (*n* = 34). In patients with severe Fisher (3–4), the WR was 1.31 (95% CI: 1.24–1.39, *p* < 0.001).

Subgroup analysis by aneurysm location revealed differential treatment effects across anatomical sites. Endovascular coiling demonstrated significant superiority in internal carotid artery locations (WR = 1.67, 95% CI: 1.47–1.90, *p* < 0.001), the anterior circulation subgroup aneurysms (WR = 1.34, 95% CI: 1.16–1.54, *p* < 0.001), and middle cerebral artery M1 segments (WR = 2.52, 95% CI: 1.82–3.50, *p* < 0.001). Middle cerebral artery M2 locations demonstrated a strong trend favoring endovascular coiling that did not reach statistical significance (WR = 2.50, 95% CI: 0.97–6.44, *p* = 0.078), likely due to limited sample size.

### Multivariable logistic regression for poor outcome

A notable finding emerged from the comparison between unadjusted and adjusted analyses. While the win ratio demonstrated favorable outcomes for endovascular therapy (WR 1.75, *p* < 0.001), this association was substantially attenuated in multivariable analysis after adjusting for clinical severity and aneurysm morphology (OR 0.55, *p* = 0.102). This discordance likely reflects unmeasured confounding by aneurysm complexity factors beyond neck diameter and maximum size.

Significant independent predictors of poor outcome were severe Hunt & Hess (OR 5.54, 95% CI: 2.74–11.21, *p* < 0.001) and severe modified Fisher (OR 3.85, 95% CI: 1.04–14.32, *p* = 0.044). Age, sex, maximum aneurysm diameter, and neck width were not significant predictors.

The logistic regression model showed good discriminatory power (C-statistic = 0.789) and excellent calibration (Hosmer-Lemeshow test *p* = 0.851), with a McFadden R^2^ of 0.206 indicating moderate fit. Only 3.6% of the observations were identified as potentially influential (Table 3).

**Table 3:** Adjusted analysis of factors associated with poor outcome (mRS >2).

Variable	aOR	CI 95%	*p*-value
Endovascular coiling vs. Open surgery	0.55	0.27–1.13	0.102
Age, per year	1.03	1.00–1.06	0.077
Male sex	0.86	0.39–1.88	0.697
Hunt & Hess ≥3	5.54	2.74–11.21	**<0.001**
mFisher ≥3	3.85	1.04–14.32	**0**.**044**
Maximum aneurysm diameter, per mm	0.93	0.83–1.04	0.176
Neck width, per mm	1.35	0.99–1.83	0.056
Model validation	**Result**		
C-statistic	0.789		
McFadden's R^2^	0.206		
Hosmer-Lemeshow	*p* = 0.873		
Influential observations	7/194 (3.6%)		

mRS, modified Rankin score; aOR, adjusted OR; CI, confidence interval; mFisher, modified fisher.

Statistically significant values (*p* < 0.05) are shown in bold.

## Discussion

Our analysis reveals a complex picture of treatment outcomes for ruptured intracranial aneurysms that requires careful interpretation. The win ratio methodology showed favorable results for endovascular coiling over surgical clipping (WR 1.75, *p* < 0.001), with consistent advantages across mortality (WR = 1.35), functional outcomes (WR = 1.53), complications (WR = 1.70), and ICU stay (WR = 1.25). These findings align with the initial safety profile reported in acute treatment studies where coiling achieves high rates of immediate clinical success (8), and with meta-analyses reporting lower risks of ischemic infarction with endovascular approaches (6, 9).

Our multivariable analysis revealed that clinical severity markers–Hunt & Hess grade (OR 5.54, *p* < 0.001) and modified Fisher scale (OR 3.85, *p* = 0.044)–emerged as the strongest predictors of poor outcome, with magnitudes substantially greater than the treatment effect. This suggests that the patient's initial clinical condition may be more determinative of short-term outcomes than the choice between coiling and clipping *per se* in this observational setting. The consistent favorable outcomes for coiling across severity subgroups suggests potential benefits in critically ill patients, as supported by Lindgren et al. (6). However, location-specific patterns merit attention. While our study showed advantages for coiling in internal carotid and anterior communicating artery locations, randomized evidence such as Darsaut et al. (10) in middle cerebral artery aneurysms suggests clipping may offer better results in specific anatomical contexts. This reinforces that treatment selection requires individualization based on aneurysm characteristics. The remarkably high win ratio in patients with mild radiographic hemorrhage (mFisher 1–2) should be interpreted cautiously as a likely statistical artifact in a small subgroup.

However, a critical methodological consideration emerges from the comparison with our multivariable analysis. While the win ratio demonstrated favorable outcomes for endovascular therapy, this association was substantially attenuated when we adjusted for clinical severity and basic aneurysm morphology in logistic regression (OR 0.55, *p* = 0.102). This discordance likely reflects important limitations in our ability to fully account for aneurysm complexity, despite including neck diameter and maximum aneurysm size in our models; factors such as aspect ratio, vessel tortuosity, incorporated branches were not available in our retrospective dataset, causing a selection bias and tempering the strength of our conclusions regarding treatment superiority.

The different conclusions from win ratio vs. multivariable analysis are not contradictory but reflect complementary perspectives. The win ratio's sensitivity to hierarchical outcomes provides valuable patient-centered insights into real-world effectiveness, while multivariable regression highlights the challenges of isolating independent treatment effects amid clinical confounding. Rather than demonstrating statistical superiority of one methodology, this comparison underscores how different analytical approaches answer different clinical questions.

Our focus on in-hospital outcomes captures the initial safety profile where coiling may offer advantages, consistent with Lee & Park (11) and Alnaami et al. (9) regarding shorter hospital stays. However, we acknowledge the well-established trade-off that this initial benefit may be counterbalanced by long-term durability concerns, as the literature consistently shows higher rebleeding risks and recurrence rates with coiling due to lower complete occlusion rates (5, 9, 12). Recent meta-analyses (6, 9, 12) confirm that clipping offers more durable occlusion and reduces retreatment needs.

Beyond the previously noted limitations of retrospective design and single-center data, our inability to fully account for aneurysm complexity through comprehensive scoring systems represents a fundamental constraint. The attenuation of the treatment effect in adjusted analyses, despite including neck diameter and aneurysm size, strongly suggests that residual confounding by unmeasured anatomical factors limits causal interpretation. Therefore, our findings should be viewed as describing associations in real-world practice rather than demonstrating treatment superiority.

## Conclusion

While hierarchical outcome analysis suggests favorable short-term results for endovascular coiling, the substantial attenuation of this association in complexity-adjusted models indicates that apparent advantages may reflect case selection patterns rather than inherent treatment superiority. Treatment decisions should continue to be individualized based on comprehensive aneurysm assessment, patient factors, and local expertise, with recognition that initial procedural advantages must be balanced against long-term durability considerations. The win ratio proved to be a valuable tool for providing a patient-centered assessment of multidimensional treatment effects.

## Data Availability

The raw data supporting the conclusions of this article will be made available by the authors, without undue reservation.
